# Trends in Nicotine Product Use Among US Adolescents, 1999-2020

**DOI:** 10.1001/jamanetworkopen.2021.18788

**Published:** 2021-08-25

**Authors:** Ruoyan Sun, David Mendez, Kenneth E. Warner

**Affiliations:** 1 Department of Health Care Organization and Policy, University of Alabama at Birmingham School of Public Health, Birmingham; 2 Department of Health Management and Policy, University of Michigan School of Public Health, Ann Arbor

## Abstract

**Importance:**

With increasing e-cigarette use among US adolescents and decreasing use of other tobacco products, it is unclear how total use of nicotine products, and its long-term health risks, have changed. The Centers for Disease Control and Prevention’s standard measure—any tobacco product use in the past 30 days—considers neither frequency of use nor product risk implications.

**Objective:**

To investigate how nicotine product use, including frequency of use, and its associated risks have changed among middle school and high school students since 1999.

**Design, Setting, and Participants:**

This cross-sectional study used data from the 1999-2020 National Youth Tobacco Survey, an in-school survey of a nationally representative sample of students in grades 6 through 12; each survey recruited between 15 000 and 36 000 participants.

**Exposures:**

Nicotine product use in the past 30 days.

**Main Outcomes and Measures:**

Use of nicotine products assessed by nicotine product days (NPDs), the number of days that the average student consumed these products in the past 30 days. Risk-adjusted NPDs account for differential long-term health risks of various products.

**Results:**

This study included 16 years of cross-sectional survey data. Each survey recruited between 15 000 and 36 000 participants in grades 6 through 12 (male students: mean, 50.4% [minimum, 48.5%; maximum, 58.4%]; mean age, 14.5 years [minimum, 14.0 years; maximum, 14.7 years]). Nationally representative cross-sectional data for high school students showed that NPDs decreased steadily from 5.6 days per month in 1999 (95% CI, 5.0-6.2 days per month) to 2.2 days per month in 2017 (95% CI, 1.9-2.6 days per month), increased to 4.6 days per month in 2019 (95% CI, 4.1-5.1 days per month), and then decreased to 3.6 days per month in 2020 (95% CI, 3.0-4.1 days per month). For a risk weight of 0.1 for e-cigarettes, compared with combustible products, risk-adjusted NPDs decreased from 2.5 days per month in 2013 (95% CI, 2.2-2.9 days per month) (prior to the popularity of e-cigarettes) to 2.0 days per month in 2019 (95% CI, 1.6-2.5 days per month) and 1.4 days per month in 2020 (95% CI, 1.0-1.8 days per month). However, with a risk weight of 1.0 for e-cigarettes (identical to that of combustible products), risk-adjusted NPDs increased to 5.3 days per month in 2019 (95% CI, 4.4-6.2 days per month) and 3.9 days per month in 2020 (95% CI, 3.1-4.7 days per month). Similar trends were found for middle school students.

**Conclusions and Relevance:**

This study suggests that NPDs represent an improvement, albeit an imperfect one, compared with any 30-day tobacco product use by incorporating the frequency of use of various products. By distinguishing products, NPDs permit consideration of the health consequences associated with different mixes of products over time. Health risks of adolescent nicotine product use could have decreased during vaping’s popularity if assessment of the long-term risks associated with vaping compared with those of smoking is low. There is a need to closely monitor youth nicotine and tobacco product use patterns.

## Key Points

**Question:**

How has exposure to nicotine products and their associated risk among US adolescents changed owing to the popularity of e-cigarettes?

**Findings:**

This cross-sectional study, which included 16 years of survey data for between 15 000 and 36 000 students in grades 6 through 12 per year, found that exposure to nicotine products, as assessed by nicotine product days, decreased prior to the popularity of e-cigarettes. This decrease slowed and then reversed owing to the upsurge of vaping; however, adjusting for differential long-term risks of nicotine products, risk-adjusted nicotine product days may have decreased if the risk associated with vaping is sufficiently low compared with that of smoking.

**Meaning:**

This study suggests that whether the health risks associated with nicotine product use among US adolescents have increased owing to the popularity of e-cigarettes depends on one’s assessment of the risks associated with vaping.

## Teaser

This cross-sectional study uses data from the National Youth Tobacco Survey to investigate how nicotine product use and its associated risks have changed among middle school and high school students since 1999.

## Introduction

In the last 2 decades, cigarette smoking has decreased steadily among US adolescents.^1,2^ In 2020, 1.6% of middle school students and 4.6% of high school students reported use of cigarettes in the past 30 days, compared with 12.8% of middle school students and 34.8% of high school students in 1999.^3,4,5^ In contrast, the popularity of e-cigarettes has increased markedly among adolescents in recent years.^6,7^ Since 2014, e-cigarettes have become the most commonly used nicotine or tobacco product among middle school and high school students.^8,9^ From 2017 to 2018, current use of e-cigarettes, defined as having vaped in the past 30 days, increased from 11.7% to 20.8% among high school students and from 3.3% to 4.9% among middle school students.^3^ The rates grew even higher in 2019, at 27.5% among high school students and 10.5% among middle school students.^10^ Vaping decreased substantially in 2020 to 19.6% of high school students and 4.7% of middle school students.^11^

Although e-cigarette use is likely substantially less hazardous to health than cigarette smoking,^12,13^ there are serious concerns about adolescent vaping. Studies have reported that use of e-cigarettes by youths who have never smoked increases the probability that they will try smoking in the next 6 to 18 months.^14,15,16,17^ Whether these findings reflect a causal relationship is debated,^18,19,20^ and it is currently unknown whether the increased likelihood of trying cigarettes will lead young people into established smoking habits.^21,22^

Most e-cigarettes contain nicotine, exposing young people to the principal chemical responsible for addiction among smokers.^23,24^ Nicotine may be especially harmful to adolescents if it adversely affects their developing brains, potentially influencing executive function and decision-making.^9,25,26^

With the increase in e-cigarette use and the decrease in use of conventional cigarettes and other tobacco products, it is unclear whether total exposure to nicotine products has changed among adolescents. Although government surveys have documented that past 30-day use of any tobacco product (including e-cigarettes) has increased during the past several years,^3,27^ they do not assess changes in total exposure to the relevant products. The Centers for Disease Control and Prevention (CDC) standard summary measure of any tobacco product (ATP) use in the past 30 days does not reflect frequency of use. Frequency varies from use of a single product on a single day to use of multiple products, including at least 1 every day. The CDC measure therefore does not account for the decrease over time in the number of days that smokers used cigarettes,^28,29^ a phenomenon that may apply to other tobacco products. More important, ATP use does not account for differential health risks associated with different products. Some products, especially cigarettes, are associated with greater health risks than others.^30^

To assess changes in exposure to nicotine products, we developed a new measure, nicotine product days (NPDs), that adjusts any past 30-day use to reflect the frequency (number of days) of individuals’ use of products during that period. Use of NPDs permits quantitative comparison of changes in exposure to various products, including cigarettes, other combustible tobacco products, smokeless tobacco, and e-cigarettes. We investigate how such exposure among middle school and high school students has changed in the last 22 years by analyzing national trends in NPDs. Because not all forms of nicotine exposure carry identical long-term health risks, we also apply product-specific risk weights to evaluate the trend in risk-adjusted NPDs during the study period.

Throughout this article, we refer to nicotine products, rather than tobacco products, because not all nicotine products are tobacco products. For instance, e-cigarettes contain no tobacco, although their nicotine is derived from tobacco. For the purposes of this study, we treat all e-cigarette use as constituting use of a nicotine product, although a minority of e-cigarettes contain no nicotine.

## Methods

### Sample

We analyzed nationally representative data on youth tobacco use from the National Youth Tobacco Survey (NYTS), a cross-sectional, school-based, self-administered survey of US middle school (grades 6-8) and high school (grades 9-12) students.^31^ For each survey, a nationally representative sample of US students was selected to participate using a 3-stage cluster sampling procedure. The US Food and Drug Administration collaborates with the CDC to administer the NYTS. Each survey asked 15 000 to 36 000 students about their tobacco-related beliefs, behaviors, and addiction, as well as their exposure to protobacco and antitobacco influences.^31^ We included all 16 NYTS surveys from 1999 to 2020. Parental consent was requested on behalf of the participants. The University of Alabama at Birmingham Institutional Review Board exempted this study from review because all data were deidentified. This study followed the Strengthening the Reporting of Observational Studies in Epidemiology (STROBE) reporting guideline.

### Measures

To assess nicotine product exposure, we evaluated past 30-day use of 9 products (cigarettes, cigars, chewing tobacco, dip, snuff, e-cigarettes, bidis, hookah, and kreteks), measuring consumption frequency as the number of days each product was used. Frequency of use of cigarettes, cigars, chewing tobacco, dip, and snuff was included in all 16 surveys, frequency of use of bidis and kreteks was included from 1999 to 2011, frequency of use of e-cigarettes was included since 2014, and frequency of use of hookah was included since 2016. Frequency data were derived from the question, “During the past 30 days, on how many days did you smoke/use [product]?” Participants entered a number from 0 to 30 to answer this question in 2019 and 2020. In prior years, they selected an answer from the following options: 0 days, 1 to 2 days, 3 to 5 days, 6 to 9 days, 10 to 19 days, 20 to 29 days, and all 30 days. In 2011, frequency categories of 3 to 5 days and 6 to 9 days were merged into 3 to 9 days for bidis and kreteks. For consistency, we converted the 2019 and 2020 data to the same categorical values as in the other surveys. For each categorical option, we took the mean value from the range to estimate the number of days of use in the past 30 days. With the exception of cigarettes and cigars, the NYTS does not assess the intensity of use of any of the products during days of use. For cigarettes and cigars, the survey asks participants how many they smoked each day on the days they smoked, with categorical response options provided. Thus, number of days constituted the 1 consistent measure of frequency available to study across all products.

### NPDs

Nicotine product days measure the number of days that an individual consumed a nicotine product in the past 30 days. We defined NPDs to be additive across different products. Their value lies between 0 and 30, where 0 stands for no product use during the month and 30 means daily product use. Nicotine product days are the sum of past 30-day use of the 9 different products, constrained to a maximum of 30. We also recorded NPDs by product type.

### Statistical Analysis

To calculate nationally representative means for NPDs among middle school and high school students, we used the svy command in Stata, version 16 (StataCorp LLC) to incorporate complex survey design using information on primary sampling unit, survey strata, and sampling weights in each year. To account for differential long-term health risks of products, we treated all combustible tobacco products as having a risk weight of 1.0, while smokeless tobacco had a risk weight of 0.1, consistent with evidence regarding the relative risk of most modern smokeless products.^32^ Combustible tobacco includes cigarettes, cigars, hookahs, bidis, and kreteks. Smokeless tobacco includes chewing tobacco, snuff, and dip. Because there is no agreed-upon risk weight for use of e-cigarettes, we varied the risk weight from 0.1 to 1.0 to demonstrate the full range of possibilities.

We considered the risk weights to include all risks associated with using a particular product as an adolescent. These included risks potentially occurring during the adolescent years, primarily addiction and harm to the developing brain, common to use of all of the products (although to different degrees), as well as long-term chronic disease risks, which are well documented and particularly concerning for cigarette smoking. The risk of vaping could include the risk that it will lead some adolescents into lifelong smoking, while the opposite is possible as well: smokers may switch to e-cigarettes, either as adolescents or later in life. Many users of nicotine products will quit using them within 1 to 2 decades of adolescence, thereby largely avoiding long-term chronic disease risks. Thus, it is virtually impossible to estimate with any precision the risk associated with the use of any product as a youth. As such, we present the full range of possible risk weights for e-cigarettes, from 0.1 to 1.0. Readers can examine results across this range to draw their own conclusions about the overall risk of contemporary patterns of product use.

We checked the robustness of our results with 2 alternative definitions of NPDs. First, we removed the 30-day cap on NPDs. The mean NPD trends between the capped and uncapped definitions are very similar, with Pearson’s correlation greater than 97%, but the uncapped NPDs reported higher values, as expected (eFigures 1 and 2 in the Supplement). In the other alternative definition of NPDs, for students using more than 1 tobacco product, only the dominant product consumption was counted. Again, the results were similar (eFigure 3 in the Supplement).

## Results

**Figure 1.**
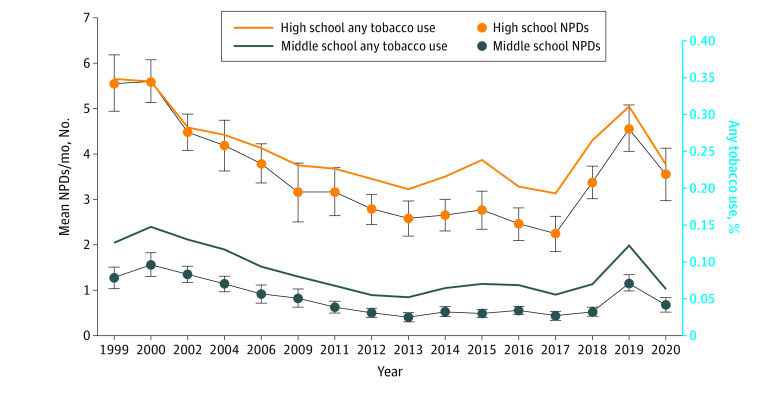
Mean Nicotine Product Days (NPDs) and Percentages of Any Tobacco Product Use Among Youths Current use of any tobacco product is defined as use of cigarettes, cigars, hookahs, e-cigarettes, smokeless tobacco, bidis, or kreteks on 1 or more days in the past 30 days. Values were reported from the National Youth Tobacco Survey, 1999-2020. Error bars indicate 95% CIs.

This study included 16 years of cross-sectional survey data. Each survey recruited between 15 000 and 36 000 participants in grades 6 through 12 (male students: mean, 50.4% [minimum, 48.5%; maximum, 58.4%], mean age, 14.5 years [minimum, 14.0 years; maximum, 14.7 years]). Using nationally representative cross-sectional data, Figure 1 presents the estimated mean NPDs among high school and middle school students from 1999 to 2020, along with estimated percentages of students who used ATP (including e-cigarettes), the CDC standard summary measure of overall product use.^3,33^ For both high school and middle school students, mean NPDs and ATP use decreased steadily through 2013, prior to e-cigarette popularity among adolescents, with NPDs decreasing more rapidly than ATP use for high school students (through 2015). This finding reflects reductions in frequency of product use by high school students captured by NPDs but not by ATP use. From 2013 to 2017, ATP use increased for 2 years and then decreased for 2 years, again for both high school and middle school students. For high school students, NPDs increased very slightly the first 2 years, while also decreasing in 2016 and 2017. For middle school students, NPDs were essentially flat. Both measures increased sharply for both high school and middle school students in 2018 and 2019 and decreased steeply in 2020. For high school students, mean NPDs started at 5.6 days per month in 1999 (95% CI, 5.0-6.2 days per month), decreased steadily to 2.2 days per month in 2017 (95% CI, 1.9-2.6 days per month), increased to 4.6 days per month in 2019 (95% CI, 4.1-5.1 days per month), and decreased to 3.6 days per month in 2020 (95% CI, 3.0-4.1 days per month). For middle school students, mean NPDs started at 1.3 days per month in 1999 (95% CI, 1.0-1.5 days per month), decreased to 0.4 days per month in 2017 (95% CI, 0.3-0.5 days per month), increased to 1.2 days per month in 2019 (95% CI, 1.0-1.3 days per month), and decreased to 0.7 days per month in 2020 (95% CI, 0.5-0.8 days per month). The estimated mean NPDs and estimated percentages of students who used ATP are highly correlated, with a correlation greater than 97% among high school students and greater than 98% among middle school students.

We also assessed mean NPDs exclusively for students who used nicotine and tobacco products (ie, excluding 0-day users). For high school students, NPDs decreased fairly steadily from 16.8 days per month in 2000 (95% CI, 16.2-17.4 days per month) to 12.1 days per month in 2015 (95% CI, 11.1-13.1 days per month), then increased to 15.3 days per month in 2020 (95% CI, 14.3-16.4 days per month). For middle school students, NPDs were reasonably steady at approximately 11.5 days per month from 2000 to 2009, decreased to 8.0 days per month in 2015 (95% CI, 7.1-8.9 days per month), and increased to 11.0 days per month in 2020 (95% CI, 9.8-12.2 days per month) (eFigure 4 in the Supplement).

**Figure 2.**
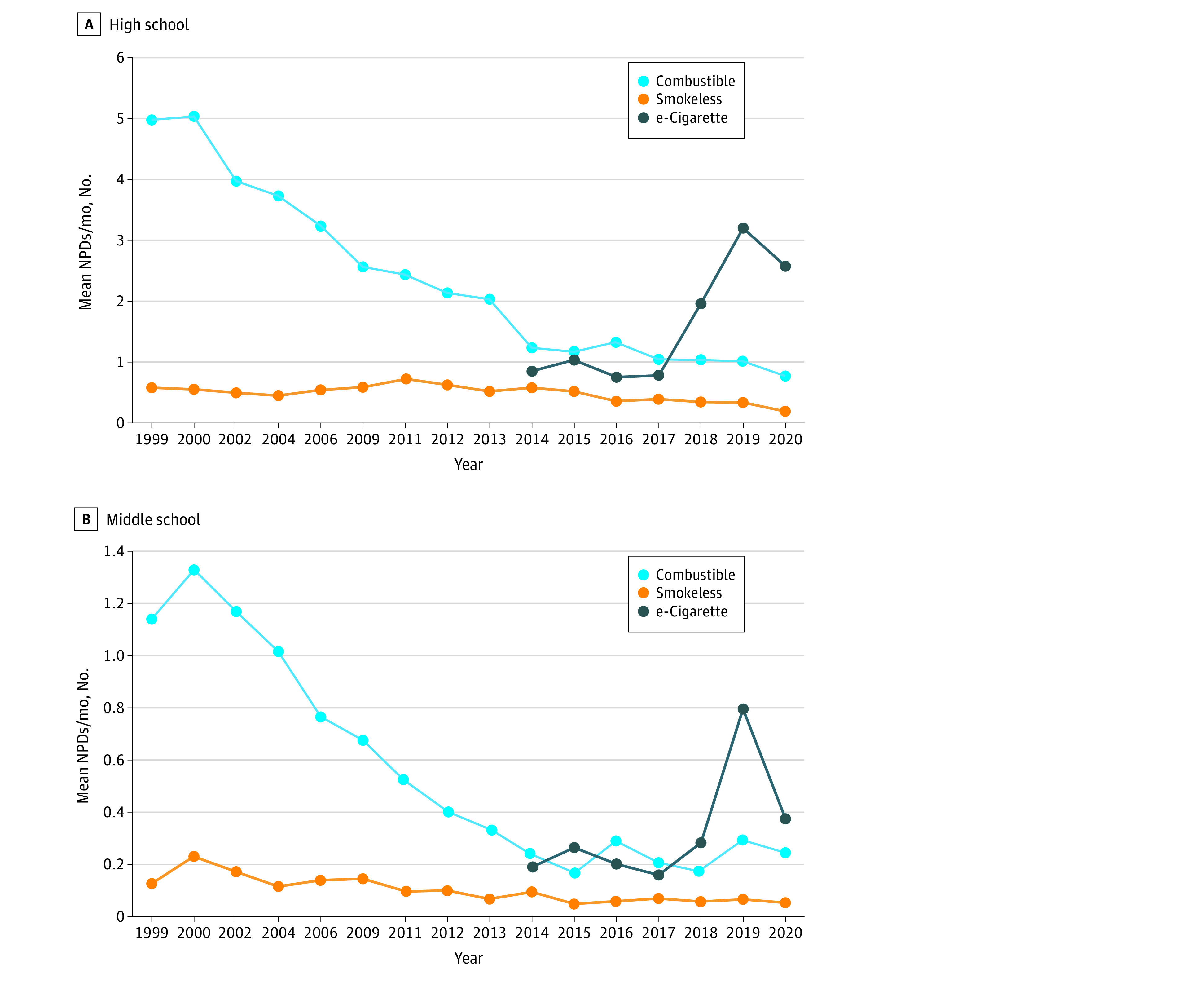
Mean Nicotine Product Days (NPDs) by Tobacco Product Type Combustible tobacco products include cigarettes, cigars, hookahs, bidis, and kreteks in available years. Smokeless tobacco products include chewing tobacco, snuff, and dip.

Figure 2 reports mean NPDs over time by product type: combustible nicotine products, smokeless tobacco, and e-cigarettes. These 3 categories exhibited substantially different trends. For high school students, the mean NPDs for combustible products has decreased sharply since 1999, from 5.0 days per month in 1999 (95% CI, 4.5-5.5 days per month) to 0.8 days per month in 2020 (95% CI, 0.6-0.9 days per month). Smokeless tobacco had relatively few days throughout the 22-year span, ranging from 0.2 to 0.8 days per month. After little change from 2014 to 2017, e-cigarette NPDs increased substantially in 2018 and 2019 and declined in 2020. Patterns were similar among middle school students.

**Figure 3.**
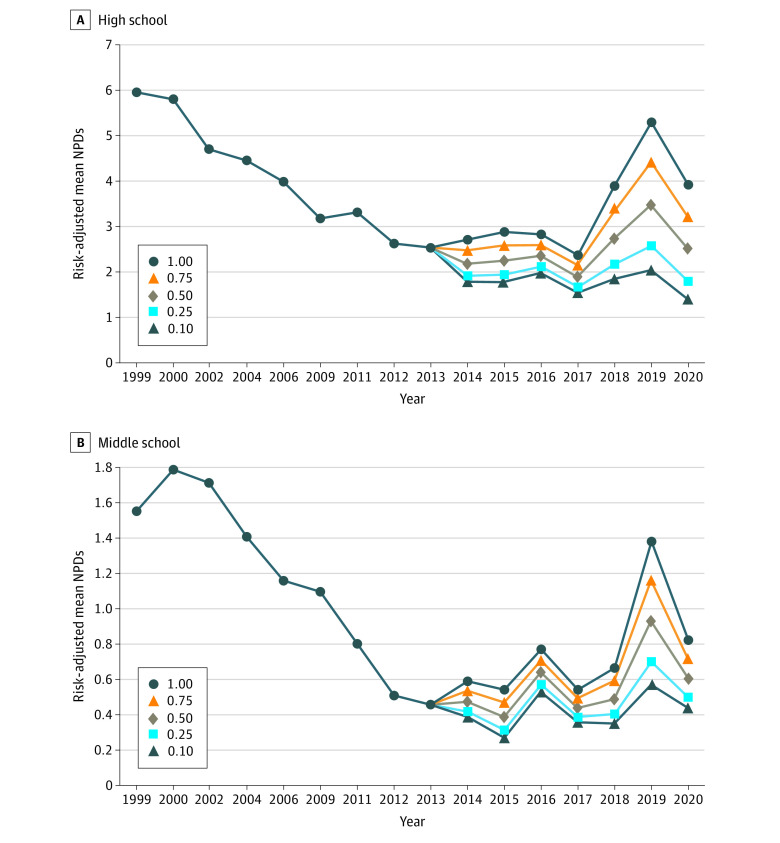
Risk-Adjusted Mean Nicotine Product Days (NPDs) Among Youths Combustible tobacco products, including cigarettes, are the reference group for risk weights, with a risk weight of 1.0. Risk weight for smokeless tobacco products is assumed to be 0.10. Risk weight for vaping is estimated at 0.10, 0.25, 0.50, 0.75, and 1.0.

Figure 3 presents mean risk-adjusted NPDs, reflecting differences in possible health risks associated with various nicotine products. For an e-cigarette risk weight of 0.1, the mean risk-adjusted NPDs among high school students has continued decreasing since 2013, from 2.5 days per month (95% CI, 2.2-2.9 days per month) to 2.0 days per month in 2019 (95% CI, 1.6-2.5 days per month) and 1.4 days per month in 2020 (95% CI, 1.0-1.8 days per month). For an e-cigarette risk weight of 1.0, identical to that of combustible products, the mean NPDs has increased steadily to 5.3 days per month in 2019 (95% CI, 4.4-6.2 days per month) and 3.9 days per month in 2020 (95% CI, 3.1-4.7 days per month). We observed similar trends among middle school students. For an e-cigarette risk weight of 0.1, the mean risk-adjusted NPDs was 0.5 days per month in 2013 (95% CI, 0.3-0.6 days per month), increasing slightly to 0.6 days per month in 2019 (95% CI, 0.4-0.7 days per month) and decreasing to 0.4 days per month in 2020 (95% CI, 0.3-0.6 days per month). With a risk weight of 1.0, the mean risk-adjusted NPDs increased to 1.4 days per month in 2019 (95% CI, 1.1-1.7 days per month) and 0.8 days per month in 2020 (95% CI, 0.6-1.1 days per month). Additional details are provided in eTables 1 and 2 in the Supplement.

## Discussion

Using our new measure of NPDs, we found substantial increases in non–risk-adjusted nicotine product exposure among adolescents since 2017 owing solely to an upsurge in the use of e-cigarettes by young people. Use of all other nicotine products has declined. Nicotine product days had decreased steadily prior to the emergence of e-cigarettes. These findings are consistent with the CDC’s measure of ATP use, although NPDs decreased more rapidly than ATP use for high school students through 2015, reflecting the reduction in the frequency of their use of tobacco products.^27^

Considering exclusively students who used tobacco products, NPDs were decreasing prior to the popularity of e-cigarettes and then increased through 2020. The increase in 2020 reflects the fact that while past 30-day prevalence of e-cigarette use decreased substantially that year for all students, among students who vaped, a larger percentage vaped more frequently than in previous years.

Although the decreased use of e-cigarettes among youths in 2020 is encouraging, the surge in 2018 and 2019 and its current prevalence remain a concern. It is impossible, however, to know the implications of current e-cigarette use for the future health of today’s adolescents. Although vaping is likely to be substantially less dangerous than smoking cigarettes,^12^ there is no definitive assessment of how much risk—or even what kinds of risk—are associated with the use of e-cigarettes. Furthermore, this undefined risk will depend to no small degree on how much and how long today’s young people use e-cigarettes, the level of nicotine concentration in e-cigarettes,^34^ the chemicals emitted by future versions of the products,^35,36^ and how many youthful e-cigarette users will transition to cigarette smoking (and how many of these individuals will then transition back to vaping).^14,15,16,17,37^

To incorporate differential health risks among products, we adjusted for product-specific risk weights to provide perspective on the risk implications of the recent increase in the use of nicotine products. The decrease in mean NPDs prior to the popularity of e-cigarettes among youths (beginning in 2014) slowed in the early years of that popularity, then increased sharply in 2018 and 2019 before decreasing again in 2020. However, if one believes, for example, that the e-cigarette risk weight should be 0.10 or 0.25, the mean risk-adjusted NPDs for high school students has continued to decline throughout the era of e-cigarette popularity. Conversely, if one believes that the e-cigarette risk weight exceeds 0.50, then risk-adjusted NPDs have increased over the past 3 years.

Most students who use e-cigarettes use them relatively infrequently. Frequent use, which is typically defined as 20 days or more in the past 30 days, is far more common among ever-smokers (current and former smokers) than among students who have never smoked and even less common among students who have never used any tobacco products.^26^ For ever-smokers who are substituting vaping for smoking, either partly or completely, vaping may be contributing little if any meaningful increase in exposure to nicotine products.^38^ Furthermore, a recent study reported that although the prevalence of nicotine product use has increased, no similar increases were found in the burden of nicotine dependence among US high school students.^39^

The significant decrease in the prevalence of youth e-cigarette use in 2020 preceded the COVID-19 pandemic. Although the precise reasons for this decrease may never be known, it could reflect responses to the EVALI (e-cigarette or vaping use–associated lung injury) scare, which was widely misinterpreted as resulting from the use of nicotine-containing e-cigarettes^40^ rather than the actual cause: vaping tetrahydrocannabinol (THC) e-cigarettes adulterated with vitamin E acetate.^41^ We will learn more from future surveys, although data for 2021 may not be directly comparable with those of previous years given that the NYTS will conduct the data collection online.

### Limitations

This study has some limitations. Two of the principal concerns expressed about vaping among youths are its potential to addict adolescents to nicotine and the possibility that nicotine may harm adolescents’ developing brains. Measuring NPDs does not attempt to assess nicotine exposure directly but rather indirectly through exposure to nicotine products. Nicotine exposure itself would be very difficult to assess, because nicotine delivery varies from one product type to another (and within product types by brand) and by user behavior (eg, frequency and depth of inhalation for combusted products and e-cigarettes). Furthermore, for most products, the NYTS does not provide daily intensity of use.

There are a few other limitations. First, the NYTS has changed use questions for a few products over time; for example, bidis and kreteks were not included in the survey after 2011. Second, we did not include pipe smoking owing to a lack of data on past 30-day use. Neither of these changes should affect our findings; all of the products dropped or excluded from our study were unpopular among adolescents (weighted prevalence, <2%).

Another limitation is that NYTS data do not make it possible to distinguish vaping nicotine from vaping THC. Another major youth survey, Monitoring the Future, found that 14% of high school seniors vaped THC in 2019.^42^ Many of these students are likely to have vaped nicotine as well.

As an in-school survey, the 2020 NYTS data were collected only in the first quarter of the year, owing to COVID-19–related school closures. This more limited data collection might affect survey findings. However, the CDC analyzed NYTS data from previous years and determined that the estimates for the entire period of data collection are virtually the same as for the truncated period in which data were collected in 2020 (Brian King, PhD, MPH, email, January 8, 2021).

## Conclusions

The results of this cross-sectional study show substantial decreases in adolescent nicotine and tobacco product use prior to the popularity of e-cigarettes (1999-2013); this decrease slowed and then reversed sharply in subsequent years. However, risk-adjusted NPDs may have continued to decline from 2014 to 2020, depending on the risk weight associated with e-cigarettes. By considering the frequency of use of nicotine products and offering a platform for contemplating the health implications of different mixes of products, we hope that NPDs represent a step forward in assessing adolescent exposure to nicotine products.

## Notes

Supplement. **eFigure 1.** Mean Nicotine Product Days (NPDs), Capped at 30 Days and Uncapped **eFigure 2.** Mean Nicotine Product Days (NPDs) by Tobacco Product Type, Capped at 30 Days and Uncapped **eFigure 3.** Mean Nicotine Product Days (NPDs) and Mean NPDs Estimated by Dominant Product Use **eFigure 4.** Mean Nicotine Product Days (NPDs) Among Nicotine Users **eTable 1.** Mean Risk-Adjusted Nicotine Product Days (NPDs) for Middle School Students **eTable 2.** Mean Risk-Adjusted Nicotine Product Days (NPDs) for High School Students
