# Supplementary material for: Trends in Nicotine Product Use Among US Adolescents, 1999-2020
Source: JAMA Netw Open. 2021 Aug 25;4(8):e2118788. doi: 10.1001/jamanetworkopen.2021.18788 (PMC8387850; doi:10.1001/jamanetworkopen.2021.18788)
Supplement: Supplement. — eFigure 1. Mean Nicotine Product Days (NPDs), Capped at 30 Days and Uncapped eFigure 2. Mean Nicotine Product Days (NPDs) by Tobacco Product Type, Capped at 30 Days and Uncapped eFigure 3. Mean Nicotine Product Days (NPDs) and Mean NPDs Estimated by Dominant Product Use eFigure 4. Mean Nicotine Product Days (NPDs) Among Nicotine Users eTable 1. Mean Risk-Adjusted Nicotine Product Days (NPDs) for Middle School Students eTable 2. Mean Risk-Adjusted Nicotine Product Days (NPDs) for High School Students [file jamanetwopen-e2118788-s001.pdf]

Supplementary Online Content

Sun R, Mendez D, Warner KE. Trends in nicotine product use among US adolescents, 1999-2020. *JAMA Netw Open.* 2021;4(8):e2118788. doi:10.1001/jamanetworkopen.2021.18788

eFigure 1. Mean Nicotine Product Days (NPDs), Capped at 30 Days and Uncapped
eFigure 2. Mean Nicotine Product Days (NPDs) by Tobacco Product Type, Capped at 30 Days and Uncapped
eFigure 3. Mean Nicotine Product Days (NPDs) and Mean NPDs Estimated by Dominant Product Use
eFigure 4. Mean Nicotine Product Days (NPDs) Among Nicotine Users
eTable 1. Mean Risk-Adjusted Nicotine Product Days (NPDs) for Middle School Students
eTable 2. Mean Risk-Adjusted Nicotine Product Days (NPDs) for High School Students

This supplementary material has been provided by the authors to give readers additional information about their work.

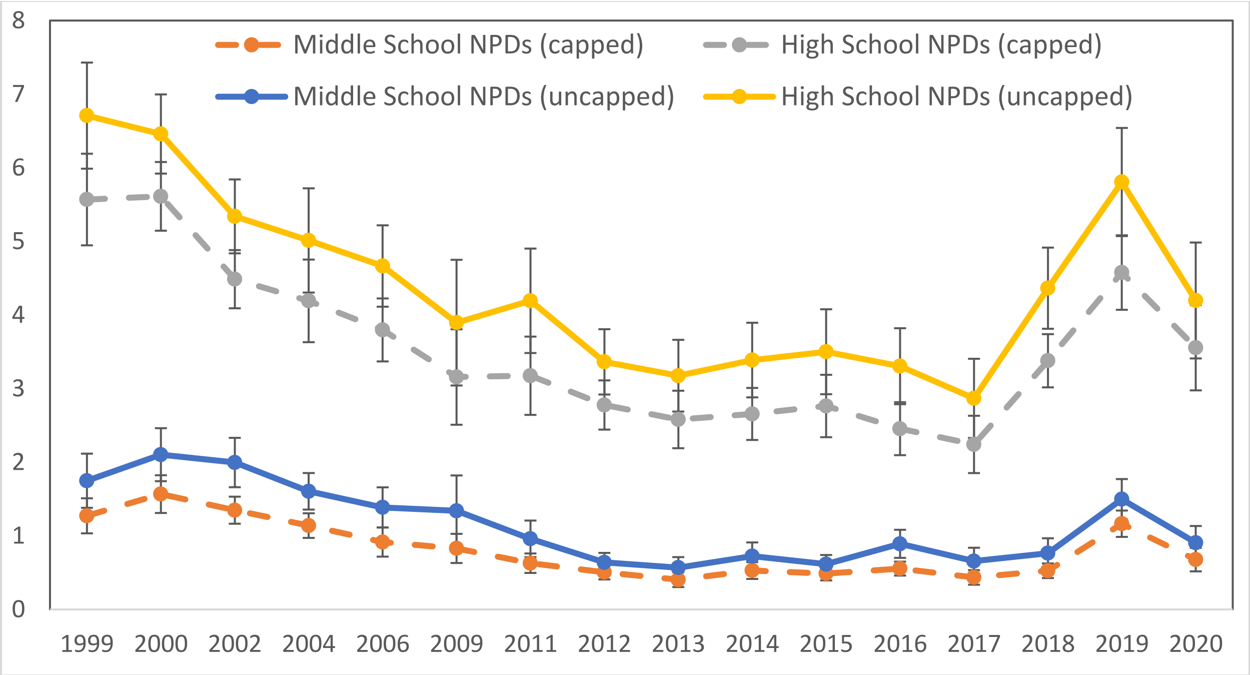

eFigure 1. Mean Nicotine Product Days (NPDs), Capped at 30 Days (dashed) and Uncapped (solid).

Mean NPDs are 98.6% correlated for middle school students and 99.2% correlated for high school students.

© 2021 Sun R et al. *JAMA Network Open.*

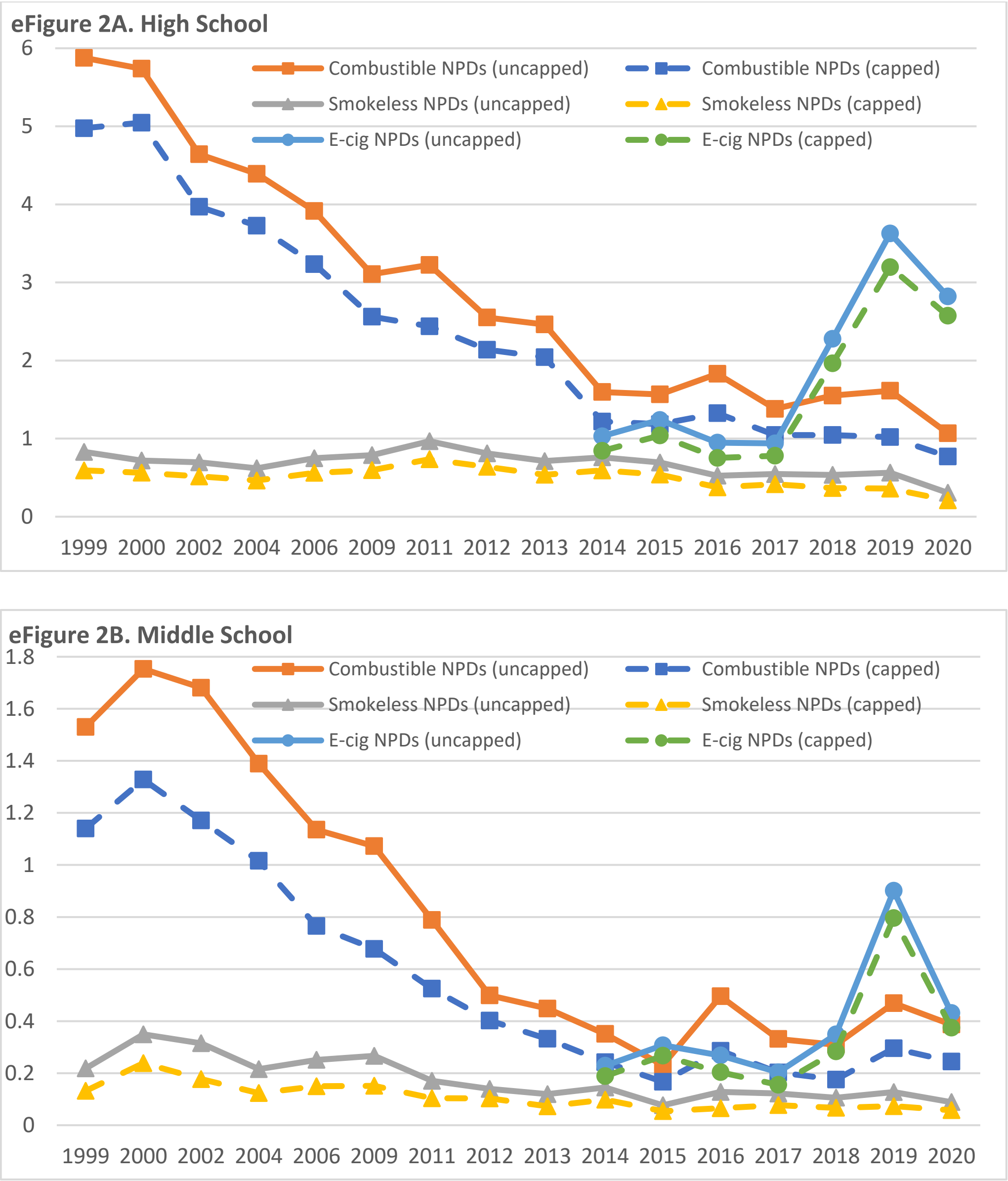

eFigure 2. Mean Nicotine Product Days (NPDs) by Tobacco Product Type, Capped at 30 Days (dashed) and Uncapped (solid).

Combustible tobacco includes cigarettes, cigars, hookahs, bidis and kreteks in available years. Smokeless tobacco includes chewing tobacco, snuff, and dip. For high school students, mean combustible NPDs (capped and uncapped) are 99.8% correlated, mean smokeless NPDs 98.6% and mean e-cig NPDs 99.9% correlated. For middle school students, these correlations are 99.4%, 97.7% and 99.9%.

© 2021 Sun R et al. *JAMA Network Open.*

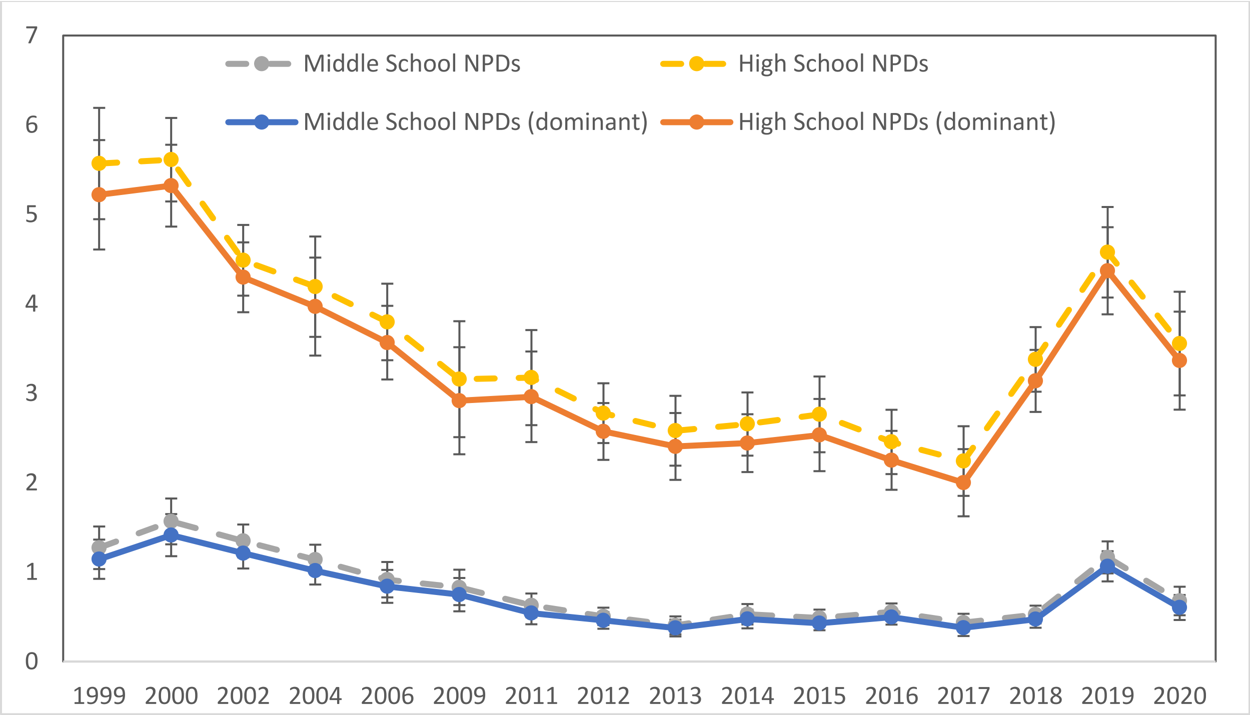

eFigure 3. Mean Nicotine Product Days (NPDs) (dashed) and Mean NPDs Estimated by Dominant Product Use (solid).

Mean NPDs for both middle school and high school are 99.9% correlated.

© 2021 Sun R et al. *JAMA Network Open.*

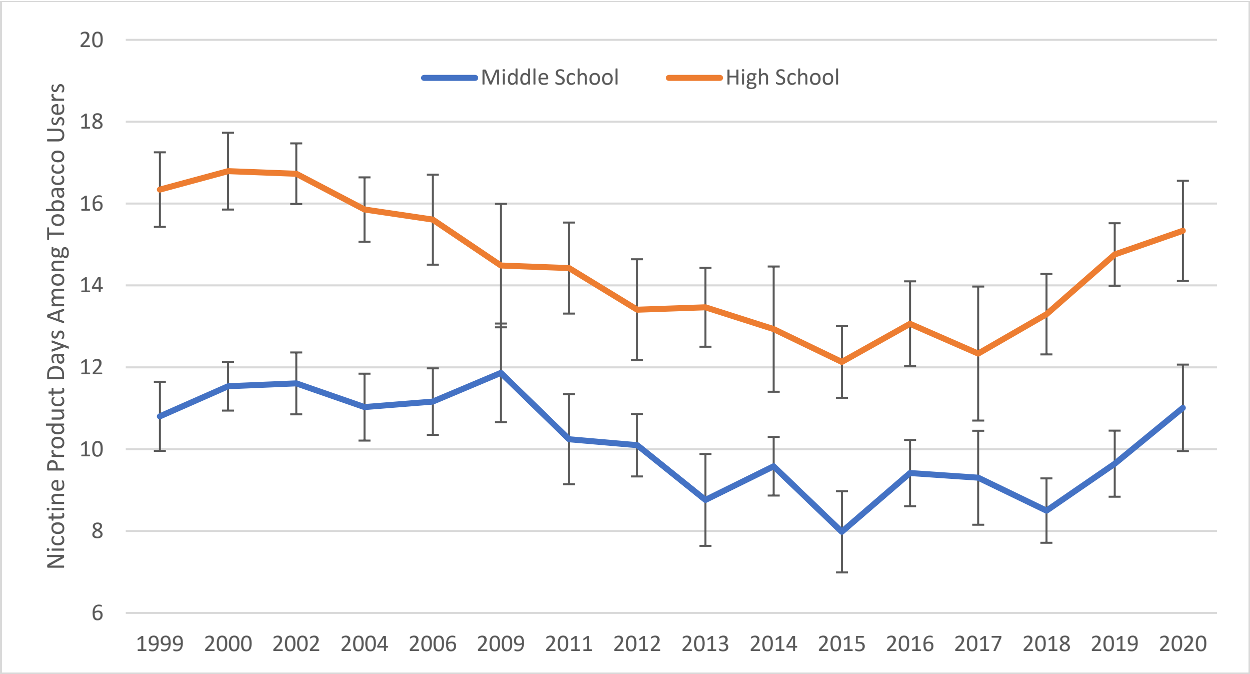

eFigure 4. Mean Nicotine Product Days (NPDs) Among Nicotine Users.

© 2021 Sun R et al. *JAMA Network Open.*

eTable 1. Mean Risk-Adjusted Nicotine Product Days (NPDs) for Middle School Students

	**Mean risk-adjusted NPDs (95% CI)**				
	E-cigarette risk weight				
Year	0.10	0.25	0.50	0.75	1.0
1999	1.55 (1.25,1.86)	1.55 (1.25,1.86)	1.55 (1.25,1.86)	1.55 (1.25,1.86)	1.55 (1.25,1.86)
2000	1.79 (1.51,2.07)	1.79 (1.51,2.07)	1.79 (1.51,2.07)	1.79 (1.51,2.07)	1.79 (1.51,2.07)
2002	1.71 (1.44,1.99)	1.71 (1.44,1.99)	1.71 (1.44,1.99)	1.71 (1.44,1.99)	1.71 (1.44,1.99)
2004	1.41 (1.19,1.64)	1.41 (1.19,1.64)	1.41 (1.19,1.64)	1.41 (1.19,1.64)	1.41 (1.19,1.64)
2006	1.16 (0.92,1.40)	1.16 (0.92,1.40)	1.16 (0.92,1.40)	1.16 (0.92,1.40)	1.16 (0.92,1.40)
2009	1.10 (0.73,1.47)	1.10 (0.73,1.47)	1.10 (0.73,1.47)	1.10 (0.73,1.47)	1.10 (0.73,1.47)
2011	0.81 (0.60,1.01)	0.81 (0.60,1.01)	0.81 (0.60,1.01)	0.81 (0.60,1.01)	0.81 (0.60,1.01)
2012	0.51 (0.41,0.62)	0.51 (0.41,0.62)	0.51 (0.41,0.62)	0.51 (0.41,0.62)	0.51 (0.41,0.62)
2013	0.46 (0.34,0.58)	0.46 (0.34,0.58)	0.46 (0.34,0.58)	0.46 (0.34,0.58)	0.46 (0.34,0.58)
2014	0.39 (0.26,0.52)	0.42 (0.28,0.56)	0.48 (0.32,0.64)	0.54 (0.36,0.71)	0.59 (0.40,0.79)
2015	0.27 (0.20,0.34)	0.32 (0.24,0.40)	0.39 (0.30,0.49)	0.47 (0.36,0.58)	0.55 (0.42,0.67)
2016	0.53 (0.40,0.67)	0.58 (0.44,0.71)	0.64 (0.49,0.79)	0.71 (0.55,0.87)	0.78 (0.60,0.95)
2017	0.36 (0.24,0.49)	0.39 (0.26,0.53)	0.44 (0.30,0.59)	0.50 (0.34,0.65)	0.55 (0.38,0.72)
2018	0.35 (0.22,0.49)	0.41 (0.26,0.55)	0.49 (0.33,0.66)	0.58 (0.40,0.77)	0.67 (0.46,0.87)
2019	0.57 (0.42,0.73)	0.71 (0.53,0.89)	0.93 (0.71,1.15)	1.16 (0.90,1.41)	1.38 (1.09,1.68)
2020	0.44 (0.30,0.58)	0.50 (0.35,0.66)	0.61 (0.43,0.79)	0.72 (0.51,0.93)	0.83 (0.59,1.06)

© 2021 Sun R et al. *JAMA Network Open.*

eTable 2. Mean Risk-Adjusted Nicotine Product Days (NPDs) for High School Students

	**Mean risk-adjusted NPDs (95% CI)**				
	E-cigarette risk weight				
Year	0.10	0.25	0.50	0.75	1.0
1999	5.96 (5.35,6.57)	5.96 (5.35,6.57)	5.96 (5.35,6.57)	5.96 (5.35,6.57)	5.96 (5.35,6.57)
2000	5.81 (5.34,6.28)	5.81 (5.34,6.28)	5.81 (5.34,6.28)	5.81 (5.34,6.28)	5.81 (5.34,6.28)
2002	4.71 (4.29,5.13)	4.71 (4.29,5.13)	4.71 (4.29,5.13)	4.71 (4.29,5.13)	4.71 (4.29,5.13)
2004	4.45 (3.85,5.06)	4.45 (3.85,5.06)	4.45 (3.85,5.06)	4.45 (3.85,5.06)	4.45 (3.85,5.06)
2006	3.99 (3.53,4.46)	3.99 (3.53,4.46)	3.99 (3.53,4.46)	3.99 (3.53,4.46)	3.99 (3.53,4.46)
2009	3.19 (2.55,3.82)	3.19 (2.55,3.82)	3.19 (2.55,3.82)	3.19 (2.55,3.82)	3.19 (2.55,3.82)
2011	3.32 (2.77,3.88)	3.32 (2.77,3.88)	3.32 (2.77,3.88)	3.32 (2.77,3.88)	3.32 (2.77,3.88)
2012	2.63 (2.28,2.99)	2.63 (2.28,2.99)	2.63 (2.28,2.99)	2.63 (2.28,2.99)	2.63 (2.28,2.99)
2013	2.53 (2.16,2.91)	2.53 (2.16,2.91)	2.53 (2.16,2.91)	2.53 (2.16,2.91)	2.53 (2.16,2.91)
2014	1.78 (1.49,2.06)	1.93 (1.61,2.25)	2.19 (1.80,2.58)	2.45 (1.99,2.90)	2.70 (2.19,3.22)
2015	1.76 (1.41,2.11)	1.95 (1.56,2.34)	2.26 (1.81,2.71)	2.57 (2.06,3.08)	2.88 (2.31,3.45)
2016	1.98 (1.57,2.38)	2.12 (1.69,2.55)	2.36 (1.88,2.83)	2.59 (2.08,3.11)	2.83 (2.27,3.39)
2017	1.53 (1.24,1.82)	1.67 (1.34,2.00)	1.91 (1.52,2.29)	2.14 (1.70,2.58)	2.38 (1.88,2.87)
2018	1.83 (1.54,2.12)	2.17 (1.84,2.51)	2.74 (2.33,3.16)	3.31 (2.82,3.81)	3.88 (3.31,4.45)
2019	2.03 (1.57,2.49)	2.58 (2.05,3.11)	3.48 (2.84,4.12)	4.39 (3.64,5.14)	5.30 (4.43,6.16)
2020	1.38 (1.02,1.75)	1.81 (1.37,2.24)	2.51 (1.95,3.07)	3.22 (2.54,3.90)	3.92 (3.12,4.72)

© 2021 Sun R et al. *JAMA Network Open.*
